# The repeat region of cortactin is intrinsically disordered in solution

**DOI:** 10.1038/s41598-017-16959-1

**Published:** 2017-12-01

**Authors:** Xiaofeng Li, Yeqing Tao, James W. Murphy, Alexander N. Scherer, TuKiet T. Lam, Alan G. Marshall, Anthony J. Koleske, Titus J. Boggon

**Affiliations:** 1 Department of Pharmacology, Yale University School of Medicine, New Haven, CT 06520 USA; 2 Department of Chemistry, Florida State University, 600 W., College Avenue, Tallahassee, FL 32306 USA; 3 Department of Cell Biology, Yale University School of Medicine, New Haven, CT 06520 USA; 4 Department of Molecular Biophysics and Biochemistry, Yale University, New Haven, CT 06520 USA; 5 Yale MS & Proteomics Resource, Yale University, New Haven, CT 06520 USA; 6 Ion Cyclotron Resonance Program, National High Magnetic Field Laboratory, 1800 E. Paul Dirac Dr., Tallahassee, FL 32310 USA; 7 Present Address: Department of Pathology, Yale University School of Medicine, New Haven, CT 06520 USA; 8 Present Address: Biopharmaceutical Analytical Sciences, Biopharm R&D, GlaxoSmithKline, 709 Swedeland Road, King of Prussia, PA 19406 USA

## Abstract

The multi-domain protein, cortactin, contains a 37-residue repeating motif that binds to actin filaments. This cortactin repeat region comprises 6½ similar copies of the motif and binds actin filaments. To better understand this region of cortactin, and its fold, we conducted extensive biophysical analysis. Size exclusion chromatography with multi-angle light scattering (SEC-MALS) reveals that neither constructs of the cortactin repeats alone or together with the adjacent helical region homo-oligomerize. Using circular dichroism (CD) we find that in solution the cortactin repeats resemble a coil-like intrinsically disordered protein. Small-angle X-ray scattering (SAXS) also indicates that the cortactin repeats are intrinsically unfolded, and the experimentally observed radius of gyration (*R*_g_) is coincidental to that calculated by the program Flexible-Meccano for an unfolded peptide of this length. Finally, hydrogen-deuterium exchange mass spectrometry (HDX-MS) indicates that the domain contains limited hydrophobic core regions. These experiments therefore provide evidence that in solution the cortactin repeat region of cortactin is intrinsically disordered.

## Introduction

Cortactin is an actin-binding protein and activator of actin branch nucleation by the Arp2/3 complex^1–4^, it also interacts with the nonreceptor tyrosine kinase Arg to regulate actin filament stability and promote actin-based protrusions in a variety of contexts^5–10^. These functions of Arg and cortactin are important for normal stabilization of dendritic spines. Defects in spine stability are associated with psychiatric disorders such as depression and schizophrenia, and neurodegenerative diseases such as Alzheimer’s Disease^11–16^. A better understanding of the structure and function of actin binding proteins (e.g. cortactin, Arg, Arp2/3) should facilitate a deeper understanding of how they regulate actin in dendritic spines and other biological contexts.

**Figure 1:**
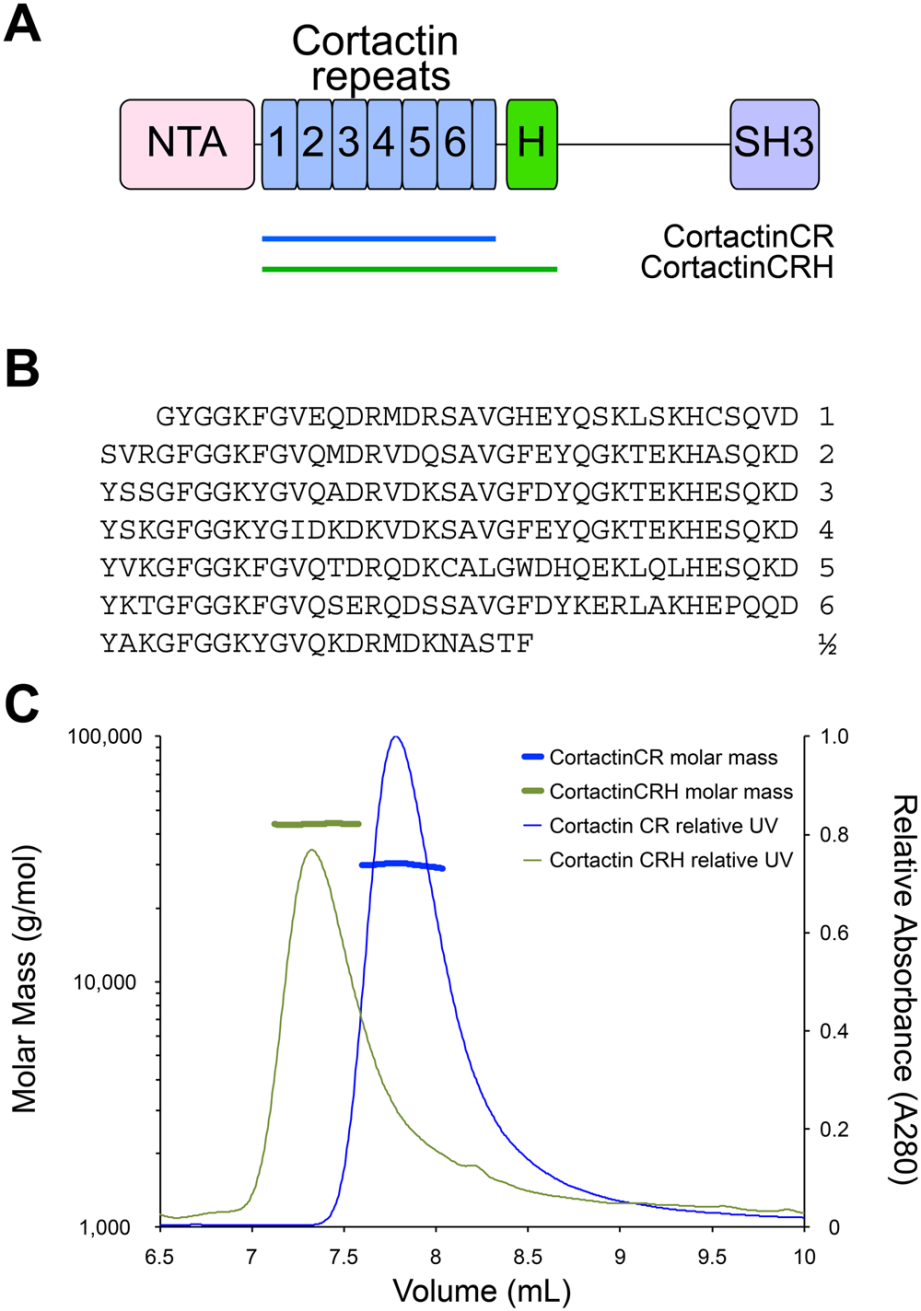
Cortactin repeats and helical domain do not homo-oligomerize. (**A**) Schematic diagram of the defined domains of cortactin. Abbreviations: NTA, amino-terminal acidic region; H, helical domain; SH3, Src-homology 3. Cortactin repeats 1 through 6 are indicated. The cortactin regions included in constructs cortactinCR and cortactinCRH are indicated. (**B**) Amino acid sequence of the 6½ cortactin repeats shown. (**C**) SEC-MALS for cortactinCRH (green) and cortactinCR (blue). Predicted molecular masses for monomeric proteins including N-terminal vector derived residues are 36.5 kDa and 27.4 kDa for cortactinCRH and cortactinCR. SEC-MALS observed experimental molecular masses are 43.9 (±1.2%) kDa and 29.9 (±1.5%) kDa for cortactinCRH and cortactinCR, respectively.

Cortactin is a multi-domain protein that contains an N-terminal acidic (NTA) domain, 6½ cortactin repeats, a helical domain that is sometimes referred to as a coiled coil domain, a flexible region that is tyrosine phosphorylated, and a C-terminal SH3 domain^17^ (Fig. 1A). The cortactin repeat region is necessary and sufficient to bind actin^18^, however, how this region folds still remains largely unknown. A series of studies have suggested that cortactin is either folded and globular^19,20^, or unfolded and extended^2,20^. These studies propose that cortactin binding to actin filaments either induces cortactin folding^21^, or does not change its secondary structure^19,20^. Therefore, to better understand the molecular function of cortactin an improved understanding of its solution state structure is required.

Here, we have conducted a series of biophysical analyses of cortactin. Size exclusion chromatography with multi-angle light scattering (SEC-MALS) shows that neither constructs of the cortactin repeats alone or the cortactin repeats together with the adjacent helical domain are able to homo-oligomerize. Circular dichroism suggests that cortactin repeats fold as a coil-like intrinsically disordered domain. Small-angle X-ray scattering indicates that the cortactin repeats are intrinsically disordered, and hydrogen-deuterium exchange mass spectrometry (HDX-MS) suggests that they contain only minimal hydrophobic core regions. Together, these experiments provide a comprehensive description of solution-state folding for the cortactin repeat domain, and allow us to define this as a coil-like intrinsically disordered domain.

## Results

### The cortactin repeat and helical domains are monomeric

Cortactin must bind to F-actin to regulate actin polymerization and branching, however, a molecular understanding of the cortactin–actin interaction, and potential conformational transitions in cortactin to allow the interaction, is lacking. The region of cortactin that is both necessary and sufficient to bind actin^18^ is termed the cortactin repeat (cortacinCR) domain. This domain contains 6½ highly similar repeats (Fig. 1A,B), and is followed by a C-terminal helical, or coiled coil, domain (Fig. 1A). Because coiled-coil domains sometimes homo-oligomerize, we wished to probe the oligomerization state of this region of cortactin so we conducted size exclusion chromatography with multi-angle light scattering (SEC-MALS) for two constructs, the cortactin repeat domain, and the cortactin repeat and helical domains (cortactinCRH) (Fig. 1C). We found that both of these domains elutes with a monomeric peak. Analysis of the SEC-MALS data indicated a molecular mass of approximately 43.9 kDa (±1.2%) for cortactinCRH and 29.9 kDa (±1.5%) for cortactinCR. The expected molecular weights for monomeric forms of these proteins (including N-terminal vector derived residues GPLGS) are 36.5 kDa and 27.4 kDa, respectively.

### Circular dichroism suggests a coil-like intrinsically disordered structure for the cortactin repeats

**Figure 2:**
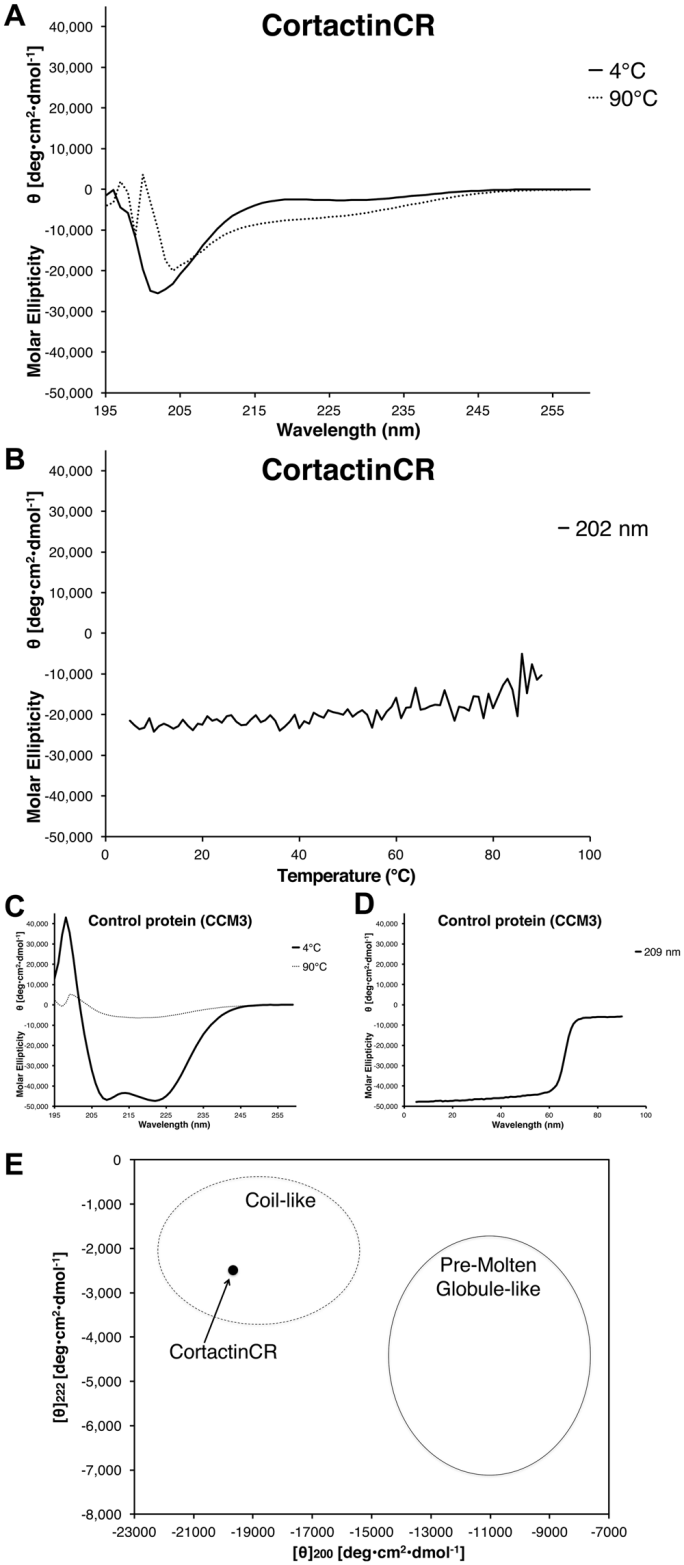
Circular Dichroism for the cortactin repeat domain. (**A**) Far UV CD spectrum for cortactinCR at 4 °C (solid line) shows a negative peak at 202 nm, but no features that could be interpreted as α-helical or β-sheet. A red shift of ~2 nm occurs on increase in temperature from 4 °C to 90 °C (dashed line). (**B**) Temperature dependence of molar ellipticity from 4 °C to 90 °C monitored at 202 nm does not show a melting point typical of folded proteins. (**C**) CD spectra for a well folded α-helical control protein, CCM3, at 4 °C and 90 °C. Secondary structure is lost at 90 °C. (**D**) Melting point analysis for CCM3 shows that this control protein melts between 60 °C and 70 °C. (**E**) Analysis of [θ]_222_ vs [θ]_200_ for cortactinCR. Plotting [θ]_222_ vs [θ]_200_ suggests that cortactinCR is falls into the coil-like unfolded protein class and not the pre-molten globule class. Analysis based on^23^.

The fold of the cortactin repeat domain remains controversial. Divergent results from the studies of this protein suggest that it is either natively unfolded^21^, or a folded protein whose secondary structure does not change upon binding to F-actin^19,20^. To settle these controversies, we first conducted circular dichroism experiments. Published circular dichroism results of different cortactin constructs came to divergent conclusions regarding the structure of the cortactin repeats^19,21^. We used circular dichroism to probe the secondary structure of cortactinCR, and compared these analyses to a well-folded control protein (CCM3) (Fig. 2). The control protein, CCM3, showed extensive secondary structure at 4 °C which was lost on heating to 90 °C (Fig. 2C). In contrast, CD spectra of the cortactinCR had a minimum negative signal at 202 nm indicating the presence of mostly random coil, consistent with natively unfolded protein. We observe a slight red shift (2 nm) and decrease in negative signal indicating minor structural changes on heating from 4° to 90 °C (Fig. 2A). Our control protein, CCM3, shows denaturation at 66.1 °C (Fig. 2D), however no such inflection point is observed for cortactinCR (Fig. 2B).

CD spectra can be deconvoluted to describe the secondary structure content of the protein of interest. We used the server, BeStSel^22^, to analyze the data from 200–250 nm for the 4 °C samples of cortactinCR and CCM3. For cortactinCR the deconvolution suggested a composition of 0% helix, 14% sheet, 13% turn and 73% other/irregular. For our control protein, CCM3 the deconvolution suggested a composition of 85% helix, 14% sheet 1% turn and 0% other/irregular, which compared favorably with crystal structures (PDB deposited structure 3L8J has a composition of 73% helix, 0% sheet, 5% turn and 22% other/irregular).

Overall, the CD data show that cortactinCR lacks significant secondary structure in solution. Furthermore, plotting [θ]_222_ vs [θ]_200_ (−2487 deg cm^2^ dmol^−1^ vs −19671 deg cm^2^ dmol^−1^) (Fig. 2E) suggests that it belongs to the intrinsic coil-like subclass of natively unfolded protein, indicating an extended conformation^23^.

### Small-angle X-ray scattering finds the cortactin repeats to be intrinsically disordered

**Figure 3:**
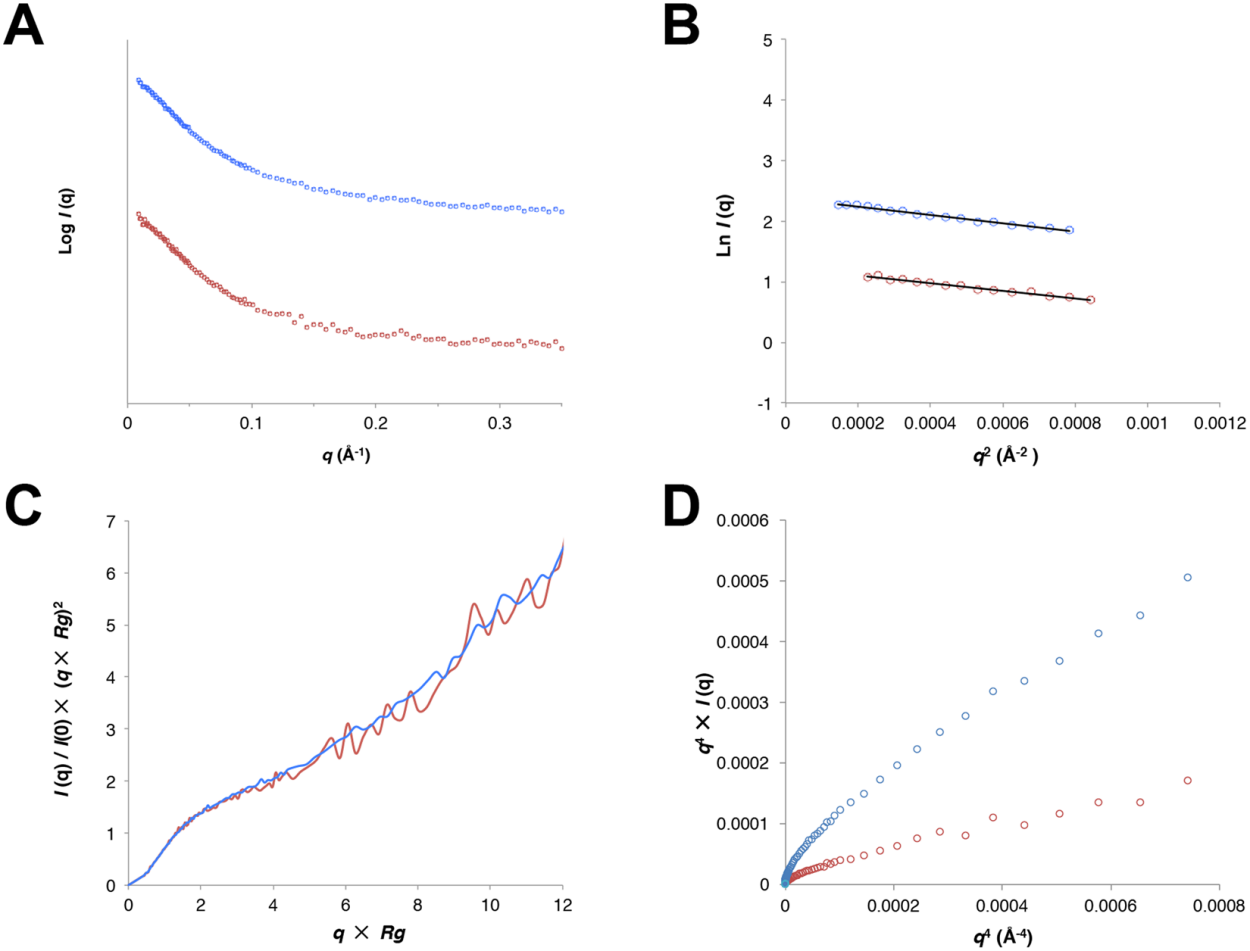
SAXS analysis for cortactin repeats (cortactinCRH). (**A**) Intensity profiles for small angle scattering of two concentrations of cortactinCRH. 1.1 mg/mL (blue) and 0.4 mg/mL (red) samples. (**B**) Linearity of the Guinier plots. Manual selection of the Guinier region is shown. (**C**) Dimensionless Kratky and (**D**) Perod-Debye plots indicate the profile of an intrinsically disordered protein.

**Figure 4:**
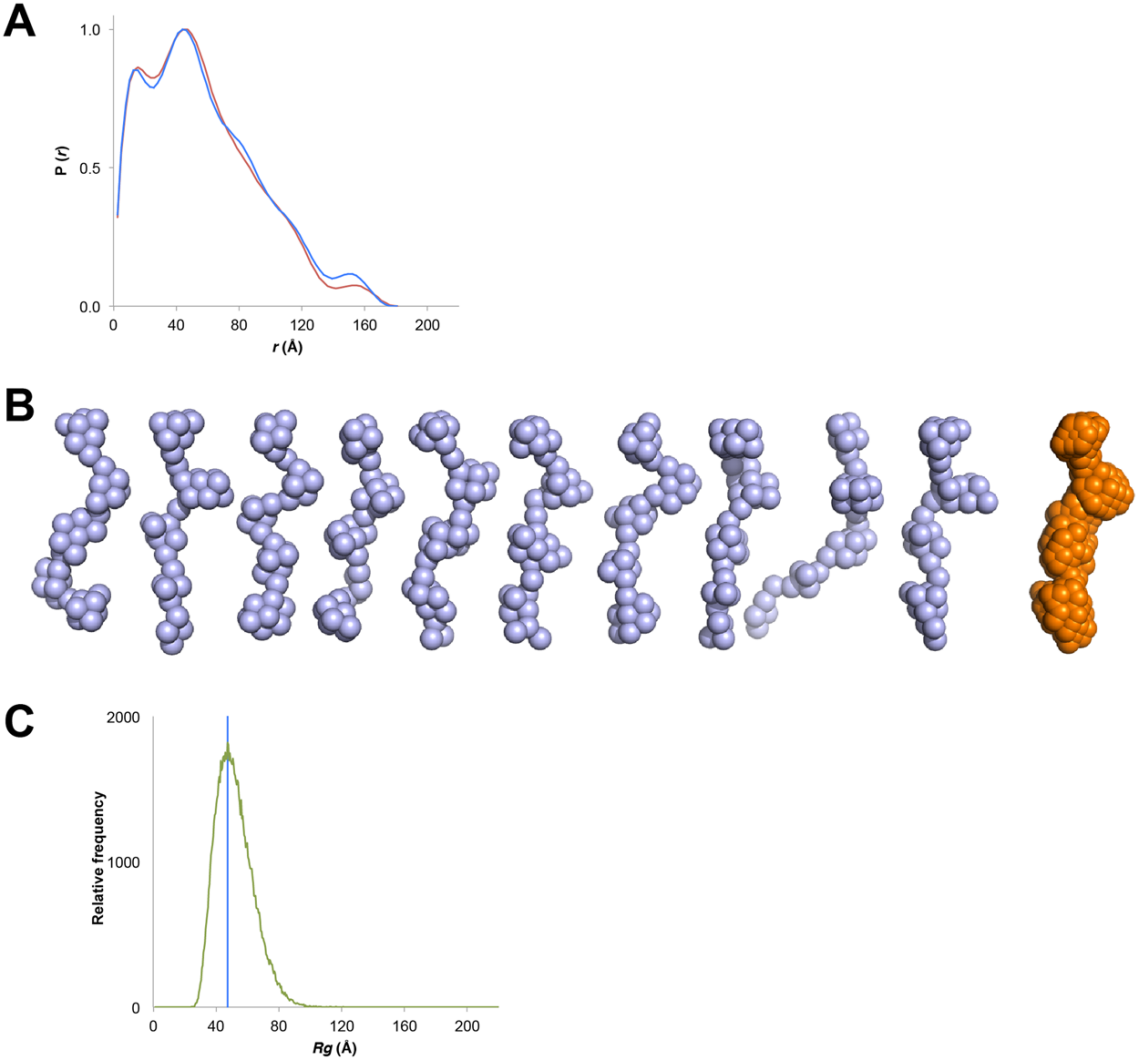
Structure of the cortactin repeats. (**A**) Normalized pair distribution function *P*(r) for cortactinCRH calculated with GNOM^44^. 1.1 mg/mL (blue) and 0.4 mg/mL (red) samples. (**B**) *Ab initio* models of CortactinCRH show extensive conformational diversity (blue). The averaged model (orange) is elongated. Models were calculated by use of DAMMIF^45^. (**C**) Calculated *R*_g_ values for the cortactinCRH sequence. Relative frequency of calculated *R*_g_ values from analysis of 100,000 molecular models of cortactinCRH as an unfolded protein based on its sequence by use of the program Flexible-Meccano^31^. Frequency of calculated *R*_g_ values (green) is compared to the observed *R*_g_ for cortactinCRH. The experimental *R*_g_ of cortactinCRH falls at the distribution peak of the calculated range.

The solution scattering properties of intrinsically disordered proteins are very different from those of folded proteins. This difference allows small-angle X-ray scattering (SAXS) to provide clear evidence of intrinsic disorder that is an orthogonal technique from circular dichroism^24–28^. Therefore, we conducted SAXS for cortactinCRH (Fig. 3A). We found no aggregation, and molecular weight estimation based on Porod volume corresponded well with those expected for a monomeric protein (Table 1), however Guinier approximations indicated a radius of gyration (*R*_g_) for this 324 amino acid protein of ~47.5 Å (Fig. 3B, Table 1), significantly larger than would be expected for a globular protein (~20 Å)^29^. Analysis of the scattering properties of cortactinCRH shows that it displays other features expected for intrinsically disordered proteins: its Kratky plot displays a monotonic increase characteristic of intrinsic disorder^24^ (Fig. 3C), and its Porod-Debye plot does not plateau as would be expected for a globular protein^30^ (Fig. 3D). We next calculated distance distribution functions P(*r*) and found an extended *D*_max_ of ~180 Å (Fig. 4A and Table 1). The molecular envelopes that we calculated for cortactinCRH were consistently elongated and conformationally diverse (Fig. 4B). Finally, to predict the overall shape and size of cortactinCRH we used the program Flexible-Meccano^31^. This is a well-validated technique that models intrinsically disordered protein structure as a random coil based on the amino acid sequence^24,31,32^. We find that the experimentally observed *R*_g_ of cortactinCRH (~47.5 Å) falls coincidently in the distribution peak (47.5 Å, 1821 occurrences) of predicted *R*_g_ values for 100,000 predicted models of an unfolded 324 amino acid peptide chain (Fig. 4C). The SAXS analysis therefore finds that cortactinCRH is intrinsically disordered as a random coil.

**Table 1:** Small-angle X-ray scattering data collection and structural statistics.

Protein	CortactinCRH	
Residue range	83–401	
Total number of amino acids (including vector-derived residues)	324	
**Data Collection Parameters**		
Beamline	NSLS-II LiX	
Beam Geometry	300 × 300 µm point source	
Detector	Pilatus 1M	
Beam Wavelength (eV)	10790	
Temperature (K)	295	
Protein concentration (mg/ml)	1.1	0.4
Q range (Å^−1^)	0.012–0.175	0.015–0.180
Number of exposures	5	5
Number of exposures averaged	5	5
Exposure time (s)	5	5
**Structural Parameters**		
*I*(0) (cm^−1^) [from *P*(r)]	10.9 ± 0.6	3.5 ± 0.5
*R*_g_ (Å) [from *P*(r)]	48.6 ± 4.3	47.1 ± 11.4
*I*(0) (cm^−1^) (from Guinier)	10.9	3.5
*R*_g_ (Å) (from Guinier)	48.1	46.7
*D*_max_ (Å)	180.4	181.0
Number of *ab initio* models calculated	10	—
Porod Volume	74948	70565
**Molecular Mass**		
Theoretical (kDa)	36.5	36.5
from Porod (kDa)	44.1	41.5
from Excluded volume (kDa)	32.5	—
Number of *ab initio* models calculated	10	—
Best model p-value (CorMap)	0.254	—
Normalized Spatial Discrepancy (NSD)	0.697 ± 0.056	—
**Software**		
Primary data reduction and averaging	pyXS	pyXS
Data processing	ATSAS	ATSAS
*Ab initio* model generation	DAMMIF	—
3D graphics representation	Pymol	—

### Hydrogen-deuterium exchange mass spectrometry shows rapid exchange for most regions of the cortactin repeats

**Figure 5:**
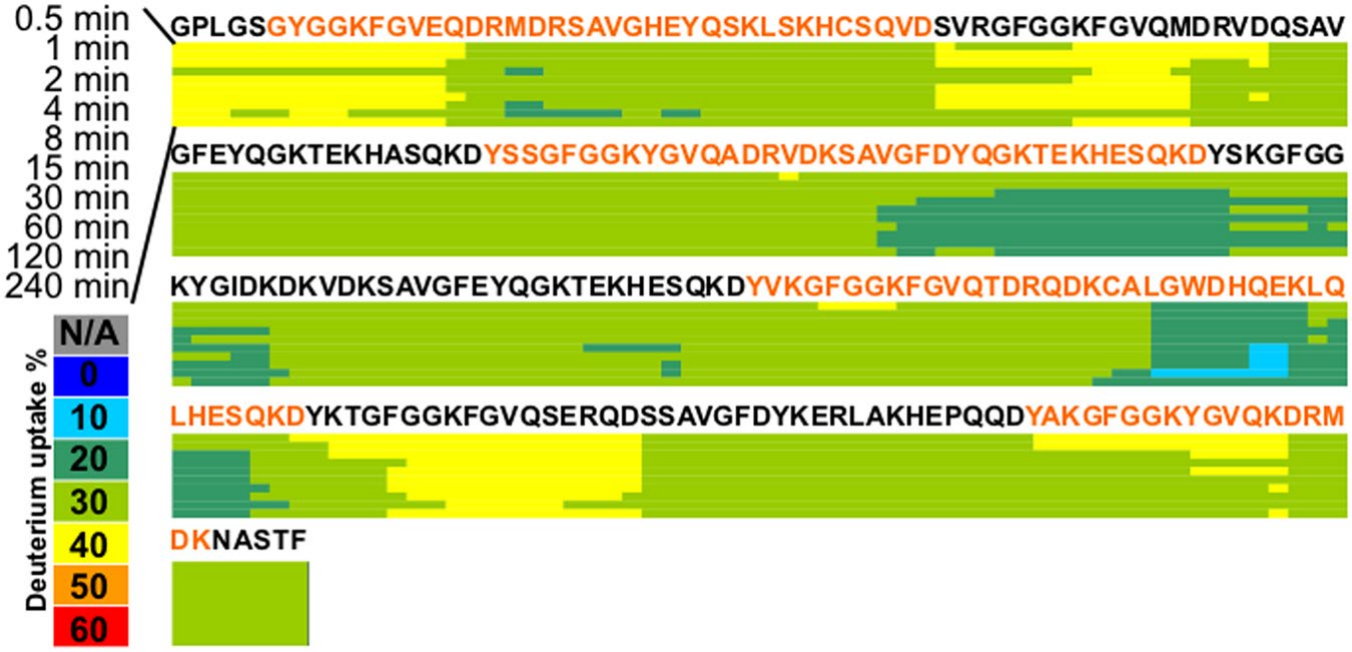
Hydrogen-deuterium exchange mass spectrometry for cortactinCR. Percentage of deuterium uptake is indicated for HDX incubation periods ranging from 30 s to 240 min. Minimal changes in deuterium uptake are observed over the time course suggesting a minimal hydrophobic core for cortactinCR, and that the protein is largely unprotected and in an unfolded state. Alternating orange and black sequences indicate cortactin repeats.

H/D exchange monitored by mass spectrometry (HDX-MS) measures the rate of replacing covalently bonded backbone amide hydrogens with deuterium atoms. Because H/D exchange depends on the solvent accessibility/hydrogen bonding of the amide hydrogen, H/D exchange as an analytical technique is a good probe for the protein conformational dynamics and interactions^33–36^. We conducted an HDX-MS time-course study for cortactinCR by incubating cortactinCR by incubating cortactinCR with D_2_O for 0, 0.5, 1, 2, 4, 8, 15, 30, 60, 120, and 240 min H/D exchange periods. For each proteolytic peptide, the percentage of D-uptake (i.e., number of deuteriums divided by the number of amide hydrogens (not counting proline(s)) after each incubation period was color-coded to produce a heat map. Examination of the cortactinCR data reveals a significant correlation of solvent exposure with the previously described CD and SAXS experiments. We find most regions of cortactinCR rapidly reached HDX saturation by the first time-point (Fig. 5), indicating that cortactinCR contains minimal hydrophobic core (unprotected) and is largely intrinsically disordered.

## Discussion

Cortactin contains 6^1/2^ cortactin repeats that form what is termed the ‘cortactin repeat domain’ (Fig. 1. Whether and how the cortactin repeats domain folds in solution has been controversial, and the literature supports two possibilities, either an extended or natively unfolded^2,21^, or a folded domain^19,20^. To resolve the question of whether the cortactin repeats are folded in solution we conducted studies based on the orthogonal biophysical techniques of circular dichroism, small-angle X-ray scattering, and hydrogen-deuterium exchange mass spectrometry. Our studies clearly demonstrate the intrinsically disordered nature of the repeat region of cortactin.

We began by probing the overall oligomerization state of the repeat region of cortactin. We were particularly interested in what is termed either the ‘coiled coil’ or ‘helical’ region C-terminal to the cortactin repeats. Coiled coil domains are mediators of homotypic or heteromeric protein-protein oligomerization (e.g.,^37^); the presence of a region with coiled coil properties would raise the question of whether or not cortactin can oligomerize through this domain. Our SEC-MALS analysis of purified constructs of cortactinCR and cortactinCRH convincingly show that there is no oligomerization (Fig. 1), so we propose that this region of cortactin be exclusively referred to as the ‘helical region’.

We next conducted circular dichroism for the cortactin repeat region of the protein (Fig. 2). This analysis clearly showed a lack of intrinsic disorder, and a complete lack of denaturation as observed by heating to 90 °C. This behavior is typical of intrinsically disordered proteins. Furthermore, our analysis suggests that the circular dichroism can classify cortactin as a coil-like unfolded protein rather than a pre-molten globule protein^23^. Pre-molten globule-like proteins contain more ordered secondary structure than the coil-like group^23^, indicating that any conformational changes of the cortactin repeats to a folded domain would be extensive, and perhaps suggesting that the cortactin repeats may not form a folded domain on binding to actin filaments.

Small-angle X-ray scattering is an orthogonal technique that can demonstrate intrinsic disorder, and for the cortactinCRH construct demonstrate a clear unfolded profile in the Kratky plot (Fig. 3C), which for folded proteins tends towards a bell-shaped distribution with a well-defined maximum^24^. Furthermore, the *P*(r) curves demonstrate an extended molecule (Fig. 4A), and our observation of *R*_g_ at ~47.5 Å matches extremely well with the predicted shape of an intrinsically disordered protein of 324 amino acids in length (Fig. 4C). The extended nature of the cortactinCRH construct in solution (*D*_max_ at ~180 Å) also correlates well with previous studies based on deep etch electron microscopy and analytical ultracentrifugation that found the full-length protein to be an extended molecule of between 220 Å and 290 Å in length^2^. When taken together, the CD and HDXMS analysis indicate that the cortactin repeats are intrinsically disordered in solution.

Overall, we have conclusively shown that the cortactin repeat domain is an intrinsically disordered in solution, however, the molecular basis of how cortactin binds to actin remains undiscovered. Low-resolution negative stain EM found that the repeats do not interact with actin filaments in an extended fashion^38^, and crosslinking suggested that the cortactin repeats may conformationally change upon binding to actin^21^. Some mapping also suggests that the fourth repeat may be required for the interaction with actin^39,40^. These important aspects of cortactin’s function remain poorly understood, therefore, we propose that future directions in the study of cortactin structure should focus on understanding the molecular basis for cortactin-actin binding.

## Methods

### Protein expression and purification

Three fragments of mouse cortactin (Uniprot: Q60598) comprising residues Gly83-Phe324 or Gly83-Thr401 of cortactin were subcloned into the pGEX6p-1 expression vector (GE), with an N-terminal glutathione S-transferase (GST) affinity tag followed by a PreScission protease site. These were transformed into *Escherichia coli* strain Rosetta(DE3) (Novagen) for expression. Production of the targeted proteins was induced by 0.2 mM isopropyl 1-thio-β-D-galactopyranoside (IPTG) at 16 °C overnight. Cells were harvested and lysed in 1x PBS buffer supplemented with protease inhibitors (Roche) and clarified supernatant was loaded onto glutathione-Separose 4B beads (GE) or Ni-NTA beads (GE). GST-cortactin was then digested with PreScission protease on-column overnight at 4 °C. The cleaved target protein was applied to a Resource S column (GE) in buffer of 20 mM MES pH 6, 5% glycerol, 1 mM DTT, and eluted with an NaCl gradient from 10 mM to 500 mM. The elution peak was loaded onto a Superdex 200 increase (GE) column. Each construct resulted in a single peak of cortactin protein. The final purified fragments of cortactin each contain N-terminal vector derived residues GPLGS followed by cortactin. The constructs are termed cortactinCR (residues Gly83-Phe324) and cortactinCRH (Gly83-Thr401).

### Size exclusion chromatography with multi-angle light scattering (SEC-MALS)

The purified proteins, cortactinCR and cortactinCRH, were analyzed by SEC-MALS by use of an in-line HPLC (Agilent Technologies 1260 Infinity), and MALS system (Wyatt DAWN HELEOS II, and OPTILAB T-rEX). Each SEC purified protein was loaded onto a WTC-300 silica-based column (Wyatt) in 1x PBS buffer supplemented with 0.02% sodium azide. For each run, a 100 µL sample at 0.6 mg/ml for cortactinCRH or 1.5 mg/mL for cortactinCR, was injected and flowrate was 0.4 mL/min with total 120 min profile. Astra chromatography software (Wyatt) was used for collecting and analyzing data.

### Circular dichroism (CD)

Purified cortactinCR was SEC purified in a buffer of 1x PBS supplemented with 5% glycerol. CD spectra were collected at 4 °C for cortactin-CR at a concentration of 10 µM by use of a Chriascan CD spectrometer (AppliedPhotophysics). Constant temperature spectra were collected at 4 °C and at 90 °C, and averages of 20 spectra calculated for each temperature. The control protein, CCM3, was purified as previously described^41^, and CD spectra were collected with the same CD protocol for purified CCM3 at a concentration of 12.5 µM. For stepped temperature ramp CD experiments a temperature range of 5 °C to 90 °C was analyzed, and the spectra repeated 3 times to average the data. The temperature-ramp experiments were conducted at 202 nM for cortactinCR and 209 nM for CCM3, the respective minima for their constant temperature spectra at 4 °C.

### Small angle X-ray scattering (SAXS)

CortactinCRH was dialyzed against 20 mM Tris pH 8, 300 mM NaCl 1 mM TCEP at final concentrations of 0.4 mg/ml and 1.1 mg/mL. X-ray scattering was conducted at the LiX beamline at the National Synchrotron Light Source II (NSLS-II) and data were collected with a Pilatus 1 M detector. Five individual 5-second exposures were collected for each concentration and for a buffer blank. Data integration, averaging, and buffer subtraction were conducted by use of pyXS^42^. Following inspection of each exposure with Primus^43^, radiation-damaged exposures were excluded. Exposures were merged together by use of pyXS and Guinier analysis was performed with Primus to calculate radius of gyration (*R*_g_). Pair distribution functions *P*(r) and forward scattering *I*(0) were calculated with GNOM^44^, and molecular weights estimated separately based on Porod volumes calculated in Primus, and excluded bead volumes of *ab initio* models from DAMMIF^45^. Dimensionless Kratky plots of *q*_2_ vs. *I*(q), in which *q* = *q*x*R*_g_ and *I*(q) = *I*(q)/*I*(0) were generated as described^30,46^. Porod-Debye plots of *q* ^4^x*I*(q) vs *q* ^4^ were generated as described^30^. The amino acid sequence of cortactinCRH was used to generate 100,000 models of cortactinCRH as a random coil type intrinsically disordered protein by use of the program Flexible-Meccano^31^. The expected *R*_g_ for a folded protein is calculated from the formula *R*_g_ = 0.395 * *N* ^3/5^ + 7.257, in which *N* is the number of residues (324 for cortactinCRH)^29^. The program Flexible-Mecanno^31^ was run by use of default options to generate 100,000 conformers of 324 amino acids.

### Hydrogen-deuterium exchange mass spectrometry (HDX-MS)

CortactinCR was analyzed by HDX-MS at the National High Magnetic Field laboratory (NHMFL) by use of on-line LC-ESI FT-ICR methods^47^. Purified cortactinCR was dialyzed into low Tris buffer (1.6 mM), 50 mM KCl, 1 mM MgCl_2_ at a concentration of 34 µM. H/D exchange was initiated when this stock was diluted to 1.6 mM in Tris buffer, 50 mM KCl, 1 mM MgCl_2_ in D_2_O (99.8 atom %) at pH meter reading 8.0. For the blank control, the sample was diluted in 1.6 mM in Tris buffer, 50 mM KCl, 1 mM MgCl_2_ in H_2_O at pH 8.0. For the zero-time control, HDX initiation and quench are performed simultaneously by adding quench buffer to D_2_O followed by sample addition. Triplicate LC/MS data were acquired after 0, 0.5, 1, 2, 4, 8, 15, 30, 60, 120, and 240 min incubation at 0.4 °C followed by quenching by addition of 25 μL of 200 mM tris(2-carboxyethyl)phosphine (TCEP), 6 M urea in 1.0% formic acid, and digestion with 25 μL of 40% (v/v) saturated protease type XIII (Sigma Aldrich, St Louis, MO) in 1.0% formic acid to yield a final pH of 2.3. Digestion proceeded for 3 min at 0.4 °C before injection for LC-MS analysis. All HDX experiments and HPLC separation were conducted at 1 °C, maintained by a MéCour Temperature Control cooling chamber (MéCour Temperature Control, LLC Groveland, MA).

After proteolysis, the CortactinCR peptide (with and without LmnA) separation and desalting were performed over a Pro-Zap Expedite MS C18 column (1.5 μm particle size, 500 Å pore size, 2.1 × 10 mm^2^; Grace Davidson, Deerfield, IL), with a Jasco high performance liquid chromatography/supercritical fluid chromatography (HPLC/SFC) system triggered by the HTC PAL autosampler (Eksigent Technologies). Peptides elute over a 2 min gradient from 2 to 95% Solvent B (Solvent A: acetonitrile/H_2_O/formic acid (4.5:95:0.5) and Buffer B: acetonitrile/H_2_O/formic acid (95:4.5:0.5)). After ionization by ESI at 3.8 kV, the sample was directed into a custom-built hybrid Velos Pro 14.5 T FT-ICR mass spectrometer (Thermo Fisher, San Jose, CA)^48^. Approximately 350 mass spectra were collected from m/z 210-1300 over a period of 6.5 min, at high mass resolving power (m/Δm_50%_ = 200,000 at m/z 400, in which Δm_50%_ is the magnitude spectral peak full width at half-maximum peak height).

After the deuterium uptake profile was analyzed for each of the peptides, a deuterium uptake “heat map” was drawn as the visual representation of the localized deuteration rate for the cortactinCR, to confirm and complement structural information discovered by other experiments. The “heat map” is drawn by summarizing deuterium uptake information for all peptides from the cortactinCR. Briefly, the deuterium uptake of each residue is calculated by averaging the deuteration levels of that residue from each overlapping peptide containing it, and the deuteration level of each residue is calculated by dividing the observed deuterium uptake by the maximum possible deuterium uptake for each peptide. Although deuterium uptake for each residue could vary across the peptide, so that this calculation does not represent an accurate extent of deuteration for each residue, this approach incorporates all available information from all overlapping peptides without introducing bias by manually selecting which peptide to display in the “heat map”.

### Data availability statement

Data and constructs will be made available upon reasonable request.
